# Expression of NDRG2 in human lung cancer and its correlation with prognosis

**DOI:** 10.1007/s12032-012-0421-7

**Published:** 2013-01-10

**Authors:** Shu-jun Li, Wen-yong Wang, Bing Li, Bei Chen, Bo Zhang, Xin Wang, Chang-sheng Chen, Qing-chuan Zhao, Hai Shi, Libo Yao

**Affiliations:** 1 State Key Laboratory of Cancer Biology, Department of Gastrointestinal Surgery, Xijing Hospital of Digestive Diseases, The Fourth Military Medical University, Xi’an, 710032 Shanxi People’s Republic of China; 2 State Key Laboratory of Cancer Biology, Department of Pathology and Pathophysiology, The Fourth Military Medical University, Xi’an, 710032 Shanxi People’s Republic of China; 3 Institute of Neurosurgery, Xijing Hospital, The Fourth Military Medical University, Xi’an, 710032 Shanxi People’s Republic of China; 4 Department of Gerontism, Guangzhou General Hospital of Guangzhou Military Command, Guangzhou, 510010 Guangdong People’s Republic of China; 5 Department of Urology Surgery, Tangdu Hospital, The Fourth Military Medical University, Xi’an, 710032 Shanxi People’s Republic of China; 6 Department of Health Statistics, The Fourth Military Medical University, Xi’an, 710032 Shanxi People’s Republic of China; 7 State Key Laboratory of Cancer Biology, Department of Biochemistry and Molecular Biology, The Fourth Military Medical University, Xi’an, 710032 Shanxi People’s Republic of China

**Keywords:** N-myc downstream–regulated gene 2, Lung cancer, Prognosis, Immunohistochemistry, UICC

## Abstract

We had reported that N-myc downstream–regulated gene (NDRG2) regulates colorectal cancer, breast cancer, clear cell renal cell carcinoma, pancreatic cancer, thyroid cancer and esophageal squamous cell proliferation, development, and apoptosis. The goal of this study was to determine the expression pattern of NDRG2 in human lung cancer and its correlation with prognosis. Immunohistochemistry, RT-PCR and western blot were used to explore the expression of NDRG2 in 185 human lung cancer patients. The correlation of NDRG2 expression with patients’ survival rate was assessed by Kaplan–Meier and Cox regression. Results showed that the expression level of NDRG2 was decreased in human lung cancer tissues, and NDRG2 was positively correlated with depth of invasion (*P* = 0.038), vascular invasion (*P* = 0.036), tumor grade (*P* = 0.039), and tumor size (*P* = 0.026). Both RT-PCR and Western blots demonstrated that NDRG2 mRNA and protein levels were lower in lung cancer compared to the adjacent normal tissue from the same individual, and NDRG2 level was negatively correlated with UICC stage. Additionally, survival time of lung cancer patients with high expression of NDRG2 was longer than those with low expression during the 5-year follow-up period (*P* = 0.001). Meanwhile, COX regression analysis indicated that low expression of NDRG2, ≥pT_3_, pM_1_, ≥pN_1_ and vascular invasion were independent, poor prognostic factors of lung cancer patients. These data showed that NDRG2 may play an important role in human lung cancer tumourigenesis, and NDRG2 might serve as a novel prognostic marker in human lung cancer.

## Introduction

Lung cancer has been one of the most common cancers in the world, and it is also the leading cause of cancer death in the urban population [1]. Conventional treatments are not efficient enough to improve the prognosis of this disease. Therefore, great efforts have been made to develop new strategies to combat lung cancer. Besides, it has long been acknowledged that the aggressive nature of human lung cancer is closely related to mutations of oncogenes as well as tumor suppressor genes and abnormalities in several growth factors and their receptors [2–4]. Based on these theories, gene therapies that can either modify the host responses to a tumor or directly inhibit tumor cell growth have attracted increasing attention and interest of researchers. To date, several molecular targets have been studied for treatment of lung cancer, including Rb1 gene and p53 gene replacement, Bcl-2 down-regulation, the FAS/CD95 receptor system and TRAIL, and inhibition of NF-kappa B [5, 6]. However, as multiple molecular alterations may be involved in the development of lung cancer [7], there is still a lot of work to be done to find more promising and effective therapeutic targets.

N-myc downstream–regulated gene family consists of NDRG1, NDRG2, NDRG3, and NDRG4, which share 57–65 % homology of amino acid sequence with each other [8]. Previously in our lab, using subtractive hybridization, human NDRG2/SYLD/KIAA1248 was identified from a normal human brain cDNA library [9]. Since then, a great many of studies have been carried out to probe into the characteristics of NDRG2. It was demonstrated that expression of NDRG2 was repressed by c-Myc at transcription level [10, 11] and up-regulated by hypoxia and nickel reagent [12–14]. Evidences from various literatures indicated that NDRG2 was involved in cellular differentiation and some human nerve system disorders [15–17]. NDRG2 expression was deficient in many kinds of tumors, such as melanoma, glioblastoma, thyroid cancer, colon cancer, and pancreatic cancer [18, 19], and NDRG2 was able to inhibit proliferation of certain tumor cells [20–22]. In view of these findings, NDRG2 was supposed to be a candidate tumor suppressor gene that may be developed to a new anti-tumor target. However, the possible role of NDRG2 in lung cancer remains to be elucidated.

The present study was conducted to explore the function of NDRG2 in the process of lung cancer development. By using immunohistochemistry, RT-PCR, and western blot, this study detected and compared the expression level of NDRG2 in 185 lung cancer tissues and normal controls and analyzed the relationship between NDRG2 level and clinical pathological information. Moreover, the relationship between expression of NDRG2 and survival time during the 5-year follow-up period was evaluated as well. These studies may provide important references for the possible role of NDRG2 in human lung cancer progression and whether NDRG2 might serve as a novel prognostic marker in human lung cancer.

## Methods

### Patients and specimens

Human lung cancer samples and their cognate normal lung tissues were obtained from 185 patients with primary lung cancer and underwent surgery in Department of Chest Surgery, Tangdu Hospital, between March 2001 and August 2003, without preoperative chemotherapy and/or radiation therapy. The collections comprised 72 squamous carcinomas (SC), 90 adenocarcinomas (AC), and 23 small-cell lung cancers (SCLC). The average age was 63.8 years (range: 27–78 years) with 64 women and 121 men. Cancer tissues, along with normal tissues that were at least 5 cm away from the cancer, were obtained from the patients. Western blot analysis was performed on fresh samples from 30 lung cancer patients. All 185 patients’ survival information of 60-month postoperative follow-up was acquired by telephone and mail. The median follow-up period was 33.6 months (range: 7–60 months). Patients’ characteristics, such as gender, age, UICC stage, vascular invasion, and tumor stage factors, were obtained from the medical records. Patient characteristics are summarized in Table 1. All resection samples were confirmed to be lung cancer by clinical pathology. At the same time, all the patients were staged based on the UICC staging system. Of the 185 patients, 34 (18.4 %) had T1-stage, 89 (48.1 %) had T2-stage, 62 (33.5 %) had T3- and T4-stage human lung cancer. The study was approved by the Human Research Committee of University, and all samples were taken with informed consent of the donors. Fresh tissues were either used immediately or stored at −80 °C for further studies.

**Table 1:** Correlation between clinicopathological characteristics and NDRG2 expression

	NDRG2(∓)	NDRG2(++)	NDRG2(+++)	*P*
Age				0.397
≤65	57 (62.0)	22 (23.9)	13 (14.1)	
>65	48 (51.6)	38 (40.9)	7 (7.5)	
Gender				0.401
Male	66 (54.5)	41 (33.9)	14 (11.6)	
Female	39 (60.9)	19 (29.7)	6 (9.4)	
pT				0.038
pT_1_	22 (64.7)	4 (11.8)	8 (23.5)	
pT_2_	41 (46.1)	39 (43.8)	9 (10.1)	
pT_3–4_	42 (67.8)	17 (27.4)	3 (4.8)	
pN				0.900
pN_0_	41 (55.4)	24 (32.4)	9 (12.2)	
pN_1_	39 (59.1)	20 (30.3)	7 (10.6)	
pN_2–3_	25 (55.6)	16 (35.6)	4 (8.9)	
pM				0.605
pM_0_	85 (55.9)	50 (32.9)	17 (11.2)	
pM_1_	20 (60.6)	10 (30.3)	3 (9.1)	
Vascular invasion				0.036
No	46 (50.0)	32 (34.8)	14 (15.2)	
Yes	59 (63.4)	28 (30.1)	6 (6.5)	
Tumor grade				0.039
1–2	43 (51.2)	25 (29.8)	16 (19.0)	
3	62 (61.4)	35 (34.6)	4 (4.0)	
Tumor size(cm)				0.026
≤3.8	45 (48.9)	34 (37.0)	13 (14.1)	
>3.8	60 (64.5)	26 (28.0)	7 (7.5)	
Histologic type				0.763
Squamous cell carcinoma	43 (59.7)	22 (30.6)	7 (9.7)	
Adenocarcinoma	50 (55.6)	30 (33.3)	10 (11.1)	
Small-cell lung cancer	12 (52.2)	8 (34.8)	3 (13.0)	

pT_1_ tumor size ≤3 cm, pT_2_ tumor size >3 cm, pT_3–4_ invasion outside of the lung

pN_0_ no lymph node metastasis, pN_1_ ipsilateral lymph node metastasis, pN_2–3_ contralateral lymphatic metastasis

pM_0_ no distant metastasis, pM_1_ distant metastasis

### Semi-quantitative RT-PCR

Total RNA was extracted from human lung cancer tissues as well as cultured cells with Trizol reagent (Invitrogen) according to manufacturer’s instructions. A total of 2-μg RNA was used for reverse transcription, and cDNA was generated and used as the template for RT-PCR analysis. GAPDH was used as an internal control to normalize variances. RT-PCR was performed using the Superscript Preamplification System (Invitrogen). PCR conditions for all of the reactions were as follows: 94 °C for 30 s, 58 °C for 30 s and 72 °C for 40 s (28 cycles). The following primers were designed using ABI Primer Express software: for NDRG2: 5′-ATGGCGGAGCTGCAGGAGGTG-3′ (forward) and 5′-AACAAGGGCCATTCAACAGGAGAC-3′ (reverse); for p53: 5′-TACTCCCCTGCCCTCAACAAGA-3′ (forward) and 5′-CTTAGCACCTGAAGGGTGAAATATTC-3′ (reverse); for GAPDH: 5′-GCCTCAAGATCAGCAAT-3′ (forward) and 5′-AGGTCCACCACTGACACGTT-3′ (reverse). The luminance was analyzed using Kodak software, and the expression level of NDRG2 was represented as follows: negative (no luminance at all); low (>20 % decrease compared with adjacent normal-appearing tissue); and high (≤20 % decrease compared with normal tissue). Each experiment was performed in triplicate.

### Western blots

The frozen tissues and cultured cells were analyzed in modified RIPA buffer (0.05 M Tris–HCl, pH 7.4, 1 % NP-40, 0.25 % Na-deoxycholate, 0.15 M NaCl, 0.001 M Na_3_VO_4_, 0.001 M EDTA and 0.5 % of protease inhibitor cocktail). The lysate was centrifuged at 10,000*g*, 4 °C for 10 min, and the supernatant was collected. Protein concentration was determined by the BCA protein assay (Pierce, Rockford, IL, USA). Proteins were separated by 10 % SDS-PAGE and were transferred to PVDF membrane. Western blot analysis was carried out using the following primary antibodies: anti-NDRG2 and anti-β-actin antibody (1:1,000; Santa Cruz Biotechnology, Santa Cruz, CA, USA), followed by incubation with horseradish peroxidase (HRP)-conjugated secondary antibody (Santa Cruz Biotechnology, Santa Cruz, CA, USA). The blots were visualized using enhanced chemiluminescence kit (Amersham Pharmacia Biotech, Arlington Heights, IL, USA) according to manufacturer’s instructions. Each experiment was performed in triplicate.

### Immunohistochemistry and immunofluorescence

Different types of lung cancer tissues as well as normal lung tissues (three samples for each type of tissue) were collected. Tissue samples were fixed with 10 % formaldehyde solution (pH 7.4) and embedded in paraffin wax. Immunohistochemistry was carried out as previously described [23]. Briefly, 4-μm-thick tissue sections were dewaxed, rehydrated, heat-induced antigen retrieved in 10-mM citrate buffer (pH 6.0) at 100 °C, and then blocked with normal goat serum for 30 min. The slides were incubated with mouse monoclonal anti-NDRG2 antibody (Tago, Burlingame, CA) at 4 °C overnight, rinsed with PBS and incubated with horseradish peroxidase-labeled goat anti-mouse secondary antibody for 60 min. NDRG2 localization was revealed using 3,3′-diaminobenzidine (DAB) as the chromogen. Negative control was performed by replacing the primary antibody with normal mouse serum. Immunofluorescence was done as previously described [24]. Texas red was used as the chromogen, and the slides were observed under a fluorescent microscope.

### Staining evaluation

For NDRG2 staining, tissue specimens were examined separately by two pathologists under double-blinded conditions without prior knowledge of the clinical status of the specimens. Any disagreement was resolved by consensus after joint review. Sections without primary antibody were used as negative controls. NDRG2 expression in Lung cancer was evaluated by scanning the entire tissue specimen under low magnification (40×) and then confirmed under high magnification (200×). An immunoreactivity score (IRS) system was applied. Expression of NDRG2 was evaluated as the percentage of positive cells and staining intensity as previously described. The percentage of positive cells was evaluated quantitatively and scored as 0 for staining of ≤1 % of total cells counted, 1 for staining of 2–25 %, 2 for staining of 26–50 %, 3 for staining of 51–75 %, and 4 for staining of >75 % of the cells examined. Intensity was graded as follows: 0, no signal; 1, weak; 2, moderate; and 3, strong staining. A total “staining score” of 0–12 was calculated and graded as negative (−, score 0–1), weak (+, score 2–4), moderate (++, score 5–8), or strong (+++, score 9–12) [17–19].

### Statistical analysis

The relationship between NDRG2 expression levels and clinicopathological factors was analyzed using the Wilcoxon–Mann–Whitney test. The overall survival time of lung cancer patients was defined as the time from the surgery to death due to cancer. The Kaplan–Meier method was used to determine the cumulative probability of survival, and data were analyzed with the logrank test. Multivariate statistical analyses were done using the Cox regression model to investigate the effects of patients’ characteristics (NDRG2 expression status, gender, age, extent of primary tumor, nodal status, vascular invasion, tumor grade, and histological type) on overall survival. A score was assigned to each variable for the Cox regression analysis. A value of *P* < 0.05 was considered statistically significant.

## Results

### NDRG2 expression is absent or decreased in primary lung cancer

**Fig. 1:**
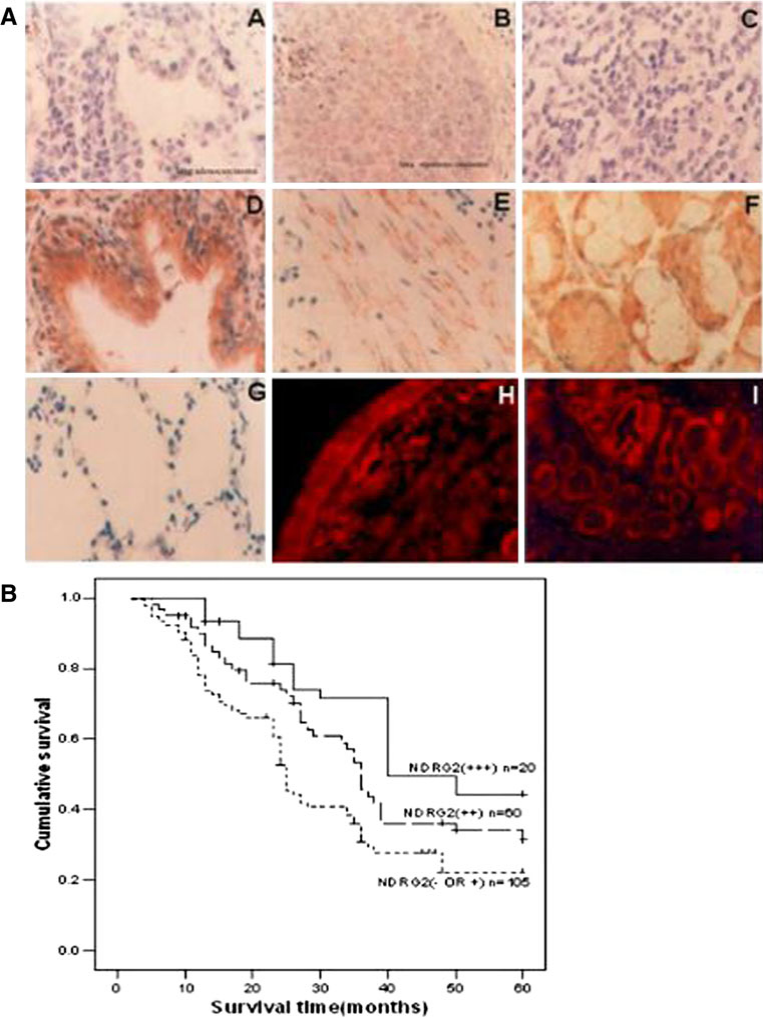
NDRG2 expression in lung cancer and normal lung tissues. **a** IHC staining showed the expression of NDRG2 in lung adenocarcinoma (*A*), squamous carcinoma (*B*) and NSCLC (*C*). IHC also showed the expression of NDRG2 in normal lung tissues, including bronchial epithelium (*D*), smooth muscle (*E*) and serous gland (*F*). Negative control (*G*). The immunofluorescent staining of NDRG2 was observed in bronchial epithelium (*H*) and submucous serous gland (*I*). Each *panel* represents the typical result of 3 samples (×200). **b** Overall survival of patients determined by the immunoreactivity of NDRG2

**Fig. 2:**
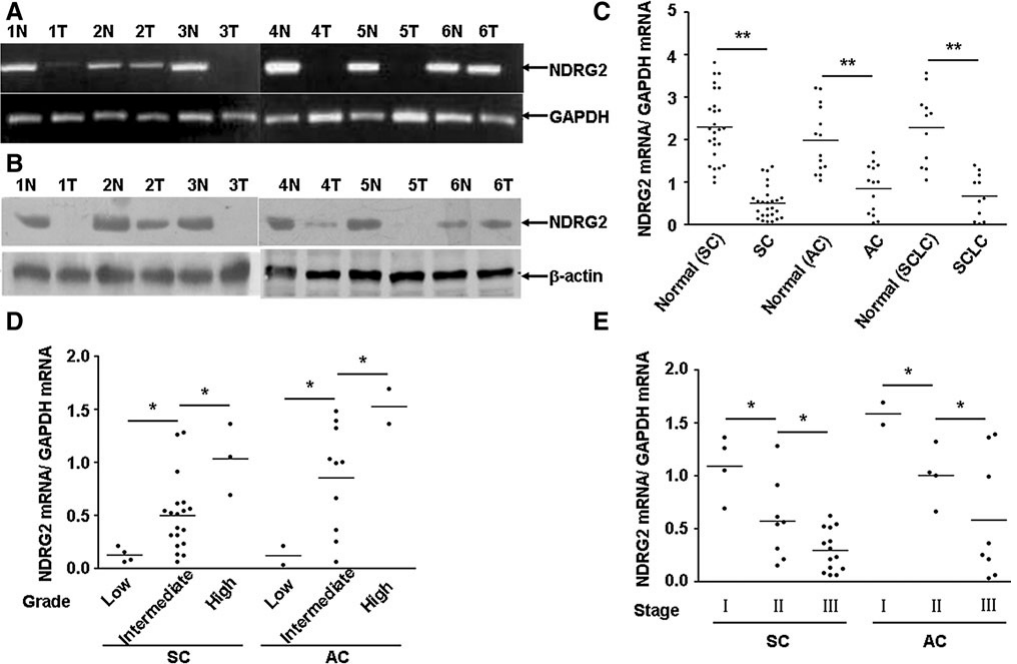
mRNA and protein level of NDRG2 in lung cancer and normal tissues, and relationship between NDRG2 level and clinical pathological parameters in lung cancer. RT-PCR (**a**) and western blot (**b**) shown that the mRNA and protein levels of NDRG2 in six lung cancer tissues (T) and cognate normal tissues (N). Three types of lung cancer (SC, AC, SCLC) exhibited significant difference of NDRG2 mRNA level in comparison with normal tissues, respectively. **c** NDRG2 mRNA level was positively correlated with tumor differentiation grade in both SC and AC (**d**) and was negatively correlated with UICC stage (**e**). *P < 0.05, **P < 0.01

NDRG2 was mainly located in the cell cytoplasm, and weak expression could be found in a few of cell nuclei. Immunohistochemistry and immunofluorescence staining of human lung cancer and match normal lung tissue specimens showed that NDRG2 was generally expressed in various normal lung tissues, including bronchial epithelium, smooth muscle, serous gland, and alveolar epithelium [see Fig. 1a (D–I)], while the staining of NDRG2 was weak or negative in SC, AC and SCLC tissues [see Fig. 1a (A–C)]. To further confirm this phenomenon, mRNA level of NDRG2 in 185 pairs of tissues containing tumor and their cognate normal counterparts from the same donor was determined by RT-PCR. The results showed that compared with normal samples, mRNA level of NDRG2 in SC, AC, and SCLC was uniformly and significantly decreased (*P* < 0.05) (see Fig. 2a, c). Protein level of NDRG2 from 30 pairs of normal and tumor specimens was also determined by western blot, and the results were consistent with that of RT-PCR analysis (see Fig. 2b).

### Relationship between NDRG2 expression and clinicopathological characteristics of lung cancer patients

The correlation between the clinicopathological characteristics and NDRG2 expression is shown in Table 1. According to the immunohistochemical results, 105 (56.76 %) of the 185 lung cancer samples were categorized as exhibiting negative or weak staining (∓). In contrast, 60 (32.43 %) and 20 (10.81 %) were scored as exhibiting moderate positive staining (++) and strong positive staining (+++), respectively. NDRG2 expression was positively correlated with depth of tumor invasion (*P* = 0.038), vascular invasion (*P* = 0.036), tumor grade (*P* = 0.039), and tumor size (*P* = 0.026). However, it was not correlated with patients’ age, gender, local lymph node metastasis, distant metastasis, and histological grade (see Table 1).

### Survival analysis

The 5-year survival rate of 185 patients was 37.8 %. The overall survival analysis using the Kaplan–Meier method revealed that the prognosis of lung cancer patients with high or moderate NDRG2 expression was significantly better than those with no or weak NDRG2 expression, and moderate expression was better than high expression (see Fig. 1b; *P* = 0.001). Multivariate analyses showed that low expression of NDRG2 (*P* < 0.001), depth of invasion ≥pT3 (*P* < 0.001), distant organ metastasis (pM1) (*P* < 0.001), regional lymph node metastasis (≥pN1) (*P* < 0.001), and vascular invasion (Yes) (*P* = 0.008) were independent, poor prognostic factors of lung cancer; However, age (≥65 years), gender (male), tumor size, tumor grade, and histological type (>G2) were not related to the prognosis of lung cancer (see Table 2).

**Table 2:** Cox multivariate analysis

Variables	Risk ratio (95 % confidence interval)	*P*
Age (>65)	1.094 (0.806–1.486)	0.565
Gender (female)	1.146 (0.755–1.741)	0.522
Primary tumor (pT_2_)	1.450 (0.964–2.182)	0.075
Primary tumor (pT_3–4_)	2.392 (1.814–3.154)	<0.001
Regional lymph node metastasis (pN_1_)	1.368 (1.033–1.811)	0.029
Regional lymph node metastasis (pN_2_)	1.825 (1.381–2.411)	<0.001
Distant metastasis (pM_1_)	2.859 (2.218–3.840)	<0.001
Vascular invasion (Yes)	1.487 (1.111–1.990)	0.008
Grade (3)	1.363 (0.868–2.140)	0.178
Size (3.8)	1.456 (0.705–3.008)	0.310
Histologic type (adenocarcinoma)	0.985 (0.703–1.381)	0.932
Histologic type (adenosquamous carcinoma)	1.417 (0.899–2.234)	0.133
NDRG2 (++)	0.641 (0.445–0.923)	0.017
NDRG2 (+++)	0.622 (0.456–0.848)	0.003

### NDRG2 level is correlated with tumor differentiation and UICC stage

According to RT-PCR results of the 185 tested cases, it was found that compared with normal tissues, 112 (60.78 %) tumor specimens showed no or low expression of NDRG2, while 73 (39.22 %) tumor tissues showed high expression of NDRG2. As shown in Fig. 2d, the mRNA level of NDRG2 was positively correlated with differentiation grade in both squamous carcinoma and adenocarcinoma; in contrast, NDRG2 level was negatively correlated with UICC stage (see Fig. 2e). No significant difference among different types of lung cancer was observed (see Fig. 2c). These results indicated that NDRG2 might be an important factor for the maintenance of normal condition in lung tissue, and its deficiency could play a part in development and progression of lung cancer.

## Discussion

Human NDRG2 cDNA was first identified from a normal human brain cDNA library by using subtractive hybridization in our lab [9], and its genomic DNA was subsequently cloned (AY028430). In our preliminary investigations, it was discovered that NDRG2 expression was ubiquitous and especially high in normal salivary gland, brain (caudate nucleus, corpus callosum, amygdala, and putaman hippocampus), skeletal muscle, and mammary gland, whereas the expression in bone marrow, testis, peripheral blood, and placenta was relatively decreased and was almost undetectable in human pancreatic cancer, hepatocellular carcinoma, thyroid cancer, colorectal cancer, and glioma [18, 20, 25–27] and some tumor cell lines, such as, human breast cancer cell line, gastric cancer cell line, and colon adenocarcinoma cell line [14, 19, 22]. It was also reported that NDRG2 was involved in many physiological and pathophysiological processes, including cell differentiation, neurodegeneration, stress responses, and carcinogenesis [15, 17]. Further studies in colon cancer and other tumors revealed that NDRG2 level was correlated with tumor differentiation and stage [19]. Together with the distinct expression patterns between normal and neoplastic tissues and cell lines, it is suggested that NDRG2 is a differentiation-related gene and might play a vital part in homeostasis.

In the present study, it has reported for the first time that NDRG2 expression was deficient in human lung cancer compared with normal lung tissues, and NDRG2 mRNA level increased with tumor differentiation grade but decreased with tumor advancement both in squamous carcinoma and adenocarcinoma. These findings suggested that NDRG2 may be an important factor for maintenance of normal condition in the lung tissue, and its deficiency could contribute to lung cancer formation and progression. The expression level of NDRG2 was closely related to the prognosis of patients with human lung cancer. The overall survival rate of patients with high or moderate NDRG2 expression was significantly better than those with none or weak NDRG2 expression, and low expression levels of NDRG2 associated with depth of invasion (≥pT3), distant organ metastasis (pM1), regional lymph node metastasis (≥pN1), and vascular invasion (Yes) were independent of each other, poor prognostic factors of lung cancer patients. In the past research, NDRG2 was shown as a tumor suppressor gene in many kinds of tumors, which promoted differentiation and apoptosis and inhibited proliferation in a variety of tumors. And the above factors could cause changes of invasion depth, vascular invasion, tumor grade, and tumor size in lung cancer. These data suggested that NDRG2 was associated with human lung cancer, and the decreased expression of NDRG2 was correlated with a worse outcome of lung cancer patients. NDRG2 could be considered as a potentially valuable prognostic indicator in patients with lung cancer. This information might be useful to clinicians in providing individualized therapy for lung cancer patients with optimal benefit.

In summary, our data showed for the first time that NDRG2 expression was decreased in human lung cancer, and the NDRG2 level was positively correlated with tumor differentiation grade, while negatively correlated with UICC stage. NDRG2 might serve as a novel prognostic marker in human lung cancer. It can also be used as an adjunct to the UICC stage system to improve prognostication for an individual patient, particularly in the first 5 years after diagnosis of lung cancer.

## Abbreviations

NDRG2: N-myc downstream-regulated gene 2
UICC: Universal Integrated Circuit Card
Rb1: Retinoblastoma 1
P53: Tumor protein 53
Bcl-2: B-Cell lymphoma 2
NF-kappa B: Nuclear factor-kappa B
SC: Squamous carcinomas
AC: Adenocarcinomas
SCLC: Small cell lung cancers
